# *In-Vitro* Anti-Proliferative and Pro-Apoptotic Properties of *Sutureja Khuzestanica* on Human Breast Cancer Cell Line (MCF-7) and Its Synergic Effects with Anticancer Drug Vincristine

**Published:** 2018

**Authors:** Saeed Esmaeili-Mahani, Mohammad Rasoul Samandari-Bahraseman, Mohammad Mehdi Yaghoobi

**Affiliations:** a *Department of Biology, Faculty of Sciences, Shahid Bahonar University of Kerman, Iran.*; b *Laboratory of Molecular Neuroscience, Neuroscience Research Center, Institute of Neuropharmacology, Kerman University of Medical Sciences, Kerman, Iran. *; c *Research Department of Biotechnology, Institute of Science and High Technology and Environmental Sciences, Graduate University of Advanced Technology, Kerman, Iran. *; d *Razi Herbal Medicines Research Center, Lorestan University of Medical Sciences, Khoramabad, Iran.*

**Keywords:** *Satureja khuzestanica* total extract, MCF-7 cell line, Apoptosis, Proliferation, Vincristine

## Abstract

*Satureja khuzestanica* Jamzad (Marzeh Khuzestani in Persian) is an endemic plant that is widely distributed in the southern part of Iran. Despite the number of papers published on this plant, no one has focused on its anticancer effects. Therefore, the present study was designed to investigate the selective cytotoxic and anti-proliferative properties of *satureja khuzestanica* total extract (SKE). MCF-7 human breast cancer cells were used in this study. Cytotoxicity of the extract was determined using MTT and neutral red assaysafter 24 h treatment period. Biochemical markers of apoptosis (caspase-3, Bax, Bcl-2) and cell proliferation (cyclin D1) were evaluated by immunoblotting. Vincristine was used to evaluate the synergic effect of extract with an anticancer drug. The data showed that treatment of cells with SKE (150 and 200 µg/mL for 24 h) significantly reduced cell viability, activated caspase 3 and increased Bax/Bcl2 ratio. In addition, cyclin D1 expression was significantly decreased in the SKE-treated cells. In addition, concomitant treatment of the MCF-7 cells with SKE and vincristine produced a potent anti-proliferative and pro-apoptotic effect compared to extract or drug alone. In conclusion, satureja extract has a potential anti-cancer property against human breast cancer cells and its combination with chemotherapeutic agent vincristine may induce cell death effectively and be a potent modality to treat this type of cancer.

## Introduction

Canceris the second leading causeofdeath in many countries (1, 2). The disease is caused by mutations in the genome during the complex process of tumorigenesis through acquisition of Hallmarks including sustaining proliferative signaling, evading growth suppressors, resisting cell death, enabling replicative immortality, inducing angiogenesis, and ultimately invasion and metastasis leading cause of death for human. Hallmarks of cancer complete multistep process of malignancy through tumorigenesis process (3). Additional mutations were gendered by genome instability and inflammation expedites clonal evolution during tomurgenesis, thereby completing malignancy steps. Inflammatory molecules released in inflammation and the reactive oxygen species are actively mutagenic for nearby cancer cells and expediting their genetic evolution toward heightening state of malignancy (3-5).

Unfortunately, the current classical treatments (surgery, chemotherapy and/or radiotherapy) are impeded by side effectssuch as development of tumor resistant, loss of appetite, nausea and vomiting, weakness and fatigue, mouth soreness, hair loss, weight gain, premature menopause, lowered resistance to infections, bleeding, and diarrhea (2). Therefore, finding novel and effective therapeutic compounds against cancer which have ability to reduce the dosage of the medicine is a current scientific challenge. 

Recently, increasing attention has been paid to naturally acquired compounds as new candidates (3). The renewed interest in natural substances has focused attention on plants used as foods, vegetables, fruits or spices, which are a rich source of bio-nutrients or bio-active phytochemical. However, more detailed studies are needed to find the safety of these compounds (4). During the recent decades, phytochemical agents in plants have been studied as an option for the treatment and prevention of cancer. Since these compounds have fewer side effects, it is a rational choice to be studied for their anti-cancer properties thereby introducing them for complementary treatment and prevention of cancer (6-8). 

*Satureja khuzestanica *Jamzad (family of Lamiaceae) grows in southern region of Iran and the people of this area use it for its analgesic and antiseptic properties (9). Previous reports demonstrated that *satureja khuzestanica* has antioxidant, anti-hyperlipidemic (10), anti-diabetic (10, 11), and anti-inflammatory effects (12-14). Antioxidant and anti-inflammatory properties of agents are involved in cancer therapy and prevention (3, 6 and^15^). It has been previously shown that carvacrolis the main components of the satureja extract (11). Notably, it has been recently reported that the plant’s main compound carvacrol has anticancer effect and is used as safe additional in food products (16, 17). 

Until now, there is no available report on the anticancer activity of total* satureja khuzestanica* on cancer cells. Therefore, the present study is an attempt towards exploring the potential anticancer activity of this plant and its interaction with anticancer drug vincristine on MCF-7 human breast adenocarcinoma cell line.

## Experimental

*Materials*

Cell culture reagents, penicillin–streptomycin and trypsin EDTA solutions and also fetal bovine serum (FBS) were obtained from Biosera Co. (Ringmer, UK). Culture flasks and dishes were acquired from SPL Lifesciences Inc. (Gyeonggi-Do, South Korea). 3-[4,5-Dimethyl-2-thiazolyl]-2,5-diphenyl-2 tetrazolium bromide (MTT) powder, neutral red, and vincristine were purchased from Sigma (St. Louis, MO, USA). Primary polyclonal anti-caspase 3 and primary monoclonal anti-beta-actin antibodies were purchased from Cell Signaling Technology, Inc. (Beverly, MA, USA). Primary polyclonal anti-Bax, primary monoclonal anti-Bcl-2 antibodies and primary monoclonal anti-cyclin D1 antibodies were obtained from Santa Cruz Biotechnology, Inc. (Santa Cruz, CA, USA).

*Preparation of planttotal extract*

An ethanolicSatureja extract was prepared in Razi Herbal Medicines Research Center (Lorestan, Iran). The healthy leaves were dried under shaded conditions, and to avoid the decomposition of chemical constituents, dried leaves were powdered and stored in clean and dry airtight containers for further studies. A sample was deposited at the herbarium of Razi Herbal Medicines Research Center. Two-hundred grams of the air-dried leaves were ground into fine powder. The powder was extracted twice; on each occasion with one liter of 80% ethyl alcohol. The collective ethanol extract was filtered, the filtrate was concentrated to dryness under reduced pressure in a rotary evaporator, and the resulting ethanolic extract was freeze-dried. It has been reported that carvacrol (78.3%), 9-Octadecenoic acid (13.5%), hexadecanoic acid (6.7%), bis (2-ethylhexyl) phthalate (1.0%), and beta-bisabolene (0.5%) were the main compositions of the satureja extract (11, 13). Aliquot portions of the total crude satureja extract were weighed and dissolved in PBS.

*Cell culture*

Human MCF-7 cancer cells were obtained from National Cell Bank of Iran (NCBI)–Pasteur Institute of Iran (Tehran, Iran). Cells were grown with Dulbecco’s modified Eagle’s medium supplemented with 10% fetal bovine serum, penicillin (100 U/mL), and streptomycin (100 μg/mL). They were maintained at 37°C under an atmosphere of 5% CO2. After two passages, the cells were plated at a density of 5000 per well in a 96-well cell culture plate for the MTT and neutral red assay. For protein extraction, cells were grown in a 6-wellcell culture plate well and permitted to attach and grow for 24 h. Then the cells were incubated in medium containing different concentration (20-200 µg/mL) of *satureja khuzestanica* total extract (SKE) for 24 h.

**Figure 1:**
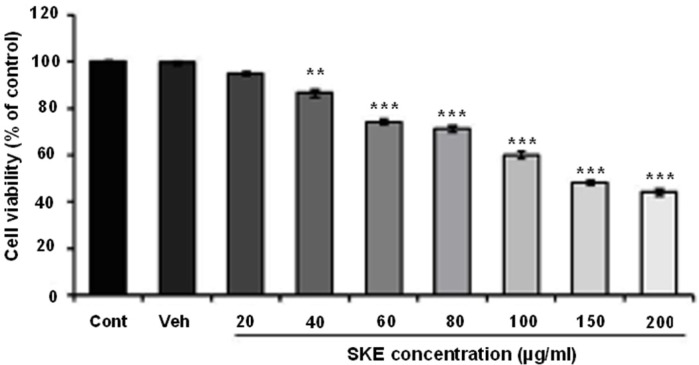
Effects of different doses of* satureja khuzestanica* total extract (SKE) on MCF-7 cancer cells viability which determined by MTT assay. Data are expressed as mean ± SEM; n = 5–6 wells for each group; **P < 0.01 and ***P < 0.001 versus control non-treated cells

**Figure 2:**
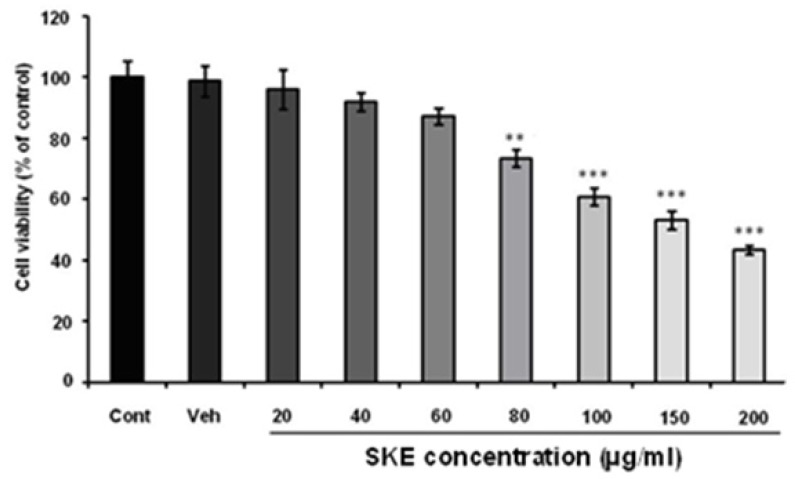
Effects of different doses of *satureja khuzestanica* total extract (SKE) on MCF-7 cancer cells viability which determined by neutral red assay. **P < 0.01, ***P < 0.001 versus control non-treated cells

**Figure 3.**
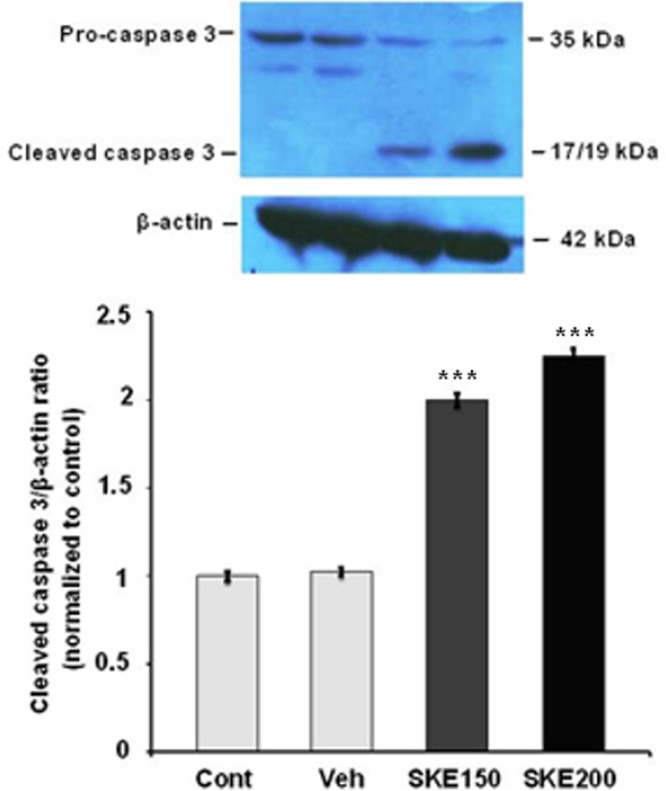
The activation of caspase-3 protein in MCF-7 cells exposed to 150 and 200 μg/mL of*satureja khuzestanica *total extract (SKE) for 24 h. Each value represents the mean ± SEM band density ratio for each group. β-actin was used as an internal control. ***P < 0.01 significantly different versus control and vehicle-treated cells

**Figure 4:**
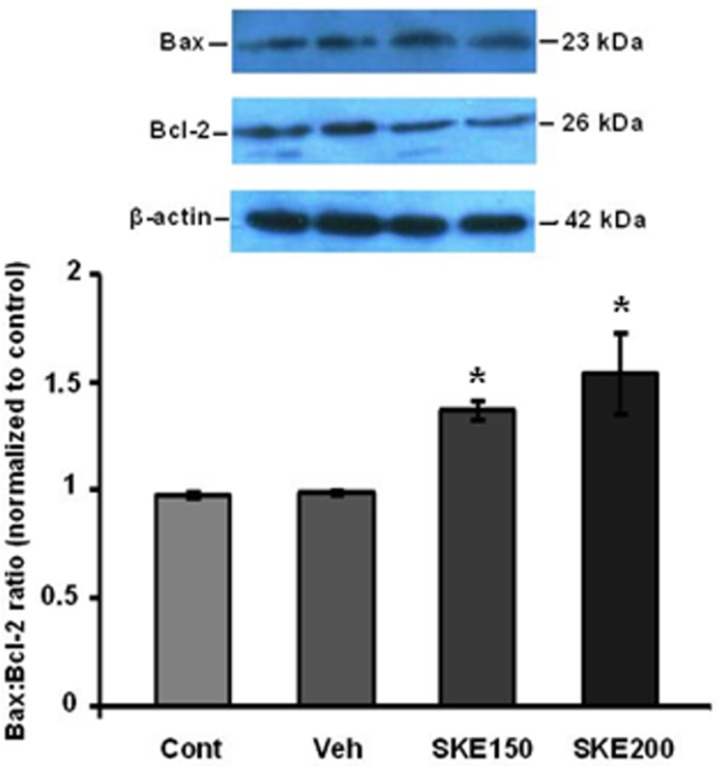
Effect of *satureja khuzestanica* extract (SKE) on levels of Bax and Bcl-2 protein expressions in MCF-7 cancer cell line. Bax and Bcl-2 protein levels were assayed by western blotting. *P < 0.05 significantly different versus control and vehicle-treated cells

**Figure 5:**
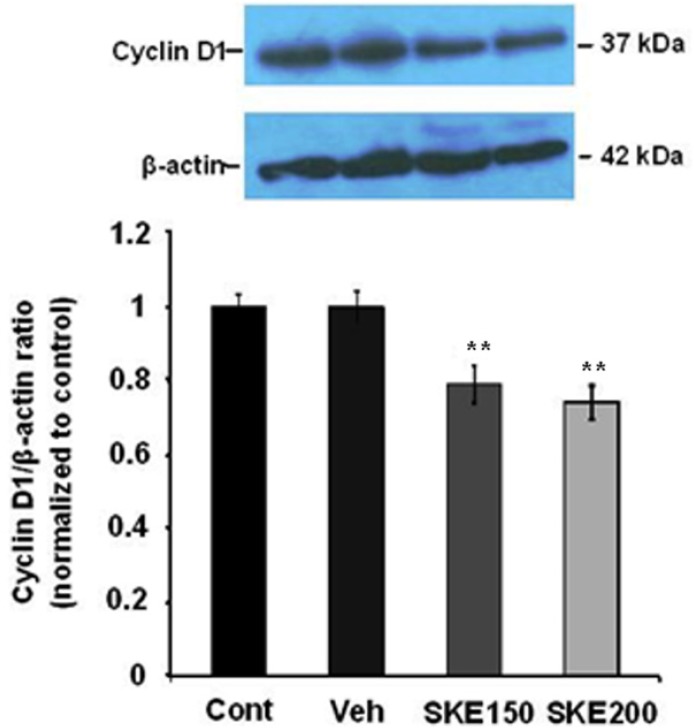
Effect of *satureja khuzestanica* total extract (SKE) on the level of cyclin D1 in MCF-7 cells. Each value represents the mean ± SEM band density ratio for each group. β-actin was used as an internal control. **P < 0.01 significantly different versus control and vehicle-treated cells

**Figure 6:**
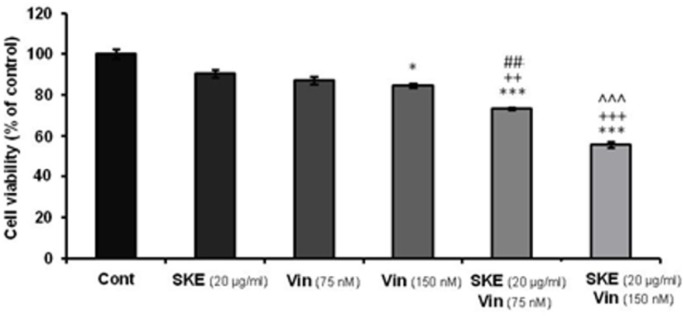
Effect of non-effective (20 μg/mL) dose of *satureja khuzestanica *total extract (SKE) alone or in combination with 75 or 150 nM vincristine on MCF-7 cell viability which determined by MTT assay. Data are expressed as mean ± SEM; n = 6 wells for each group; *P < 0.05, ***P < 0.001 versus control cells. ++*P *< 0.01 and +++*P *< 0.001 versus cells that had SKE alone. ##*P *< 0.01 versus 75 nM vincristine. **^^^***P *< 0.001 versus cells that had 150 nM vincristine alone

**Figure 7:**
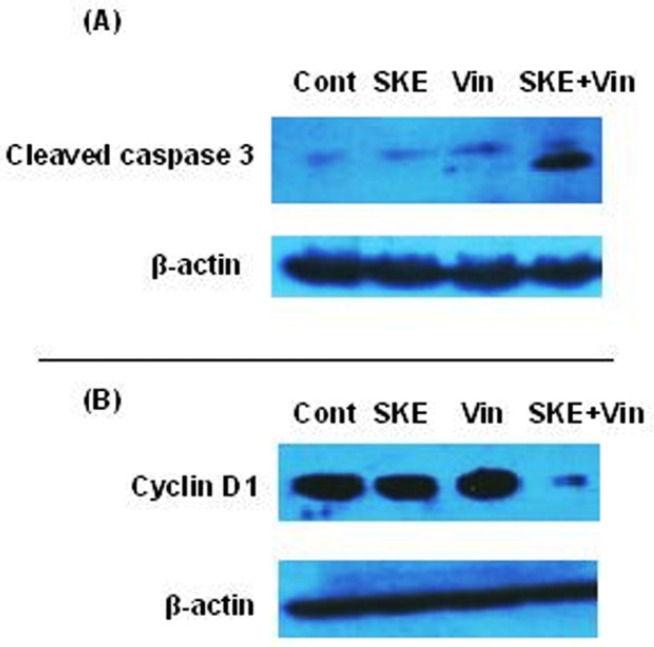
Effect of sub-effective doses of *sutureja khuzestanica* total extract (SKE, 20 µg/mL) and vincristine (Vin, 150 nM) alone or in combination on the levels of activated caspase 3 (A) and cyclin D1 (B) in MCF-7 cancer cells. β-actin was used as an internal control for loading

*Cell viability analysis*

*MTT assay*

Cellular viability was assessed by reduction of 2-(4, 5-dimethylthiazol-2-yl)-2, 5-diphenyltetrazolium bromide (MTT) to formazan (18). MTT was dissolved in PBS and added to the culture at a final concentration of 0.5 mg/mL. After an additional 2 h of incubation at 37°C, the media was carefully removed, 100 mL DMSO was added to eachwell, and the absorbance (OD) values were determined by spectrophotometry at 490 nm with an automatic microplate reader (Eliza MAT 2000, DRG Instruments, GmbH, Marburg, Germany). The results are expressed relative to the control value.

*Neutral red assay*

The neutral red assay has been used extensively for *in-vitro* assessment of cytotoxicity of infectious agents, food additives, and pharmaceuticals. This assay is based on the incorporation of neutral red (3-amino-7dimethyl-1-2-methylphenazine hydrochloride) into the lysosomes of viable cells after being incubated with test agents (19). Neutral red (4mg/mL) was diluted 1:100 into medium and incubated overnight at 37 °C and centrifuged before use. Two-hundred µL of prepared neutral red solution was added to each well and the cells were incubated at 37 °C for 3 h. After that the cells were rapidly washed with a solution of 1% calcium chloride and 0.5% formaldehyde. The dye is then extracted from the intact and viable cell with a solution of 1% acetic acid and 50% ethanol. Subsequently, absorbance (OD) values were measured by spectrophotometry at 540 nm. Results were expressed as percentages of control.

*Immunoblot analysis*

MCF-7 cells were homogenized in ice-cold buffer containing 10 mM Tris–HCl (pH 7.4), 1 mM EDTA, 0.1% SDS, 0.1% Na-deoxycholate, 1% NP-40 with protease inhibitors (1 mM phenyl methyl sulfonyl fluoride, 2.5 µg/mL of leupeptin, 10 µg/mL of aprotinin) and 1 mM sodium orthovanadate. The homogenate was centrifuged at 14000 × g for 15 min at 4 °C. The resulting supernatant was retained as the whole cell fraction. Protein concentrations were measured using the Bradford-based protein assay (Bio-Rad Laboratories GmbH, Muenchen, Germany) and equal amounts of protein (40 µg) were resolved electrophoretically on a 9% or 12% SDS-PAGE gel and then transferred to nitrocellulose membranes (Hybond ECL, GE Healthcare Bio-Sciences Corp. NJ, USA). After overnight blocking at 4 °C with 5% non-fat dried milk in Tris-buffered saline with Tween 20 (blocking buffer, TBS-T, 150 mM NaCl, 20 mM Tris–HCl, pH 7.5, 0.1% Tween 20), the membranes were probed with rabbit monoclonal antibody to caspase-3 (Cell Signaling Technology, USA, 1:1000 overnight at 4 °C), Bax (∆ 21): sc-6236, Bcl-2 (C-2): sc-7382, cyclin D1 (H-295): sc-753 (Santa Cruz, USA, 1:1000) for three h at room temperature. After washing in TBS-T (three times, each time 5 min), the blots were incubated for 60 min at room temperature with a horseradish peroxidase-conjugated secondary antibody (1:15000, GE Healthcare Bio-Sciences Corp. NJ, USA). All antibodies were diluted in blocking buffer. The antibody-antigen complexes were detected using the ECL system and exposed to Lumi-Film chemiluminescent detection film (Roch, Germany). Lab Work analyzing software (UVP, UK) was used to analyze the intensity of the expression. β-actin immunoblotting (antibody from Cell Signaling Technology, INC. Beverly, MA, USA; 1:1000) was used to control for loading. The immunoblot experiments for each protein were performed 3-4 independent times.

*Statistical analysis*

The results are expressed as mean ± SEM. The differences in mean cell viability assays between experimental groups were determined by one-way ANOVA, followed by Tukey test. The values of caspase 3, Bax, Bcl-2, cyclin D1, and β-actin band density were obtained from band densitometry. These values were expressed as tested proteins/β-actin ratio for each sample. The averages for different groups were compared by ANOVA and followed by Tukey test. *P *< 0.05 was considered significant.

## Results

*Effect of satureja khuzestanica total extract (SKE) on MCF-7 proliferation*.

To evaluate the effect of SKEon MCF-7 viability and proliferation, MTT and neutral red assays were recruited. The cells were treated with different concentration of SKE (20-200 µg/mL) for a 24 h period. MTT assay showed that SKE had a concentration dependent inhibitory effect on cell viability and it also killed about 50% of cells at doses of 150 and 200 µg/mL (Figure 1). Furthermore, cytotoxic effect of SKE was also observed in neutral red assay (Figure 2). SKE treatment in doses of 80, 100, 150, and 200 µg/mL significantly elicited cell damage after 24 h (Figure 2). SKE (20 µg/mL) had nosignificant effect on MCF-7 cells in both viability tests and was used for evaluation of its synergic effect with anticancer drug vincristine.

*Effect of satureja khuzestanica extract (SKE) on expression of molecular markers of apoptosis (Bax, Bcl-2,* and* caspase 3) in MCF-7 cancer cells****.***

For evaluation of the effect of SKE on induction of MCF-7 cells apoptosis, the level of activated caspase 3 and Bax/Bcl-2 ratio were evaluated by immunoblotting. As it is shown in Figure 3, the effective doses of extract in cell viability tests (150 and 200 µg/mL) significantly increased cleaved caspase3in a concentration dependent manner (Figure 3). 

Furthermore, western blotting analysis showed that the ratio of Bax/Bcl-2 was also significantly increased in SKE-treated cells (Figure 4).

*Effect of satureja khuzestanica extract (SKE) on cyclin D1 protein level as a marker of cell proliferation in MCF-7 cancer cells* ***.***

To examine the potential mediators of SKE-induced cell damage, we analyzed cyclin D1 protein as a marker of cell proliferation. The cells were exposed to 150 and 200 µg/mL of SKE (the most effective doses in MTT and neutral red assays) for 24 h. The amount of cyclin D1 protein levels in SKE-treated MCF-7 cells was found to be decreased (*P *< 0.001) compared to those in the cells treated with control medium (Figure 5).

*Effect of SKE in combination with vincristine on MCF-7 proliferation*

MTT assay data showed that SKE in combination with vincristine displayed synergic effects on MCF-7 cell viability (Figure 6). The sub-effective doses of vincristine (75 and 150 nM) accompanied with SKE (20 µg/mL) could produce significant toxic effect compared to drugs alone (Figure 6).

*Western blot analysis of cleaved caspase 3 and cyclin D1 in MCF-7 cells treated with satureja khuzestanica extract (SKE) and vincristine.*

To examine the synergic effect of SKE and vincristine on the up-regulation of activated caspase 3 and down-regulation of cyclin D1, the cells were exposed to control, low effective dose of SKE (20 µg/mL) and vincristine (150 nM) alone or in combination for 24 h. The blots showed that SKE plus vincristine treatment markedly increased activated caspase 3 (Figure 7A) and decreased Cyclin D1 protein expression (Figure 7B) in MCF-7 cells compared to the cells that had each drug alone.

## Discussion

Despite progress in understanding of the tomurigenesis and molecular methods and techniques to treat cancer (3, 19), chemotherapy is still considered as one of the most important methods in treating cancer patients. Since one of the problems of chemotherapy is having side effects and toxicity on other normal cells, reducing dose of this medicines with increase in sensitivity of cancer cells to low doses can reduce side effects and enhance its effectiveness (6, 7). One of the methods to reduce the dose of these medicines is combination of different medicines to increase their effects in order to decrease their usage. Herbal agents known as chemoprevention- used in dietary as a spice, flavoring, or vegetables have anti-cancer properties and these agents prevent cancer by several mechanisms preventing tumorigenesis process. Such natural compounds that have few side effects can be considered as rational options for combination with anti-cancer medicines. However, natural products require precise and scientific experimental testing as well as clinical trials before they can expect widespread choice in the management of disease.

The data indicated that SKE has anticancer properties on MCF-7 cancer cells and shows synergic effect in combination with vincristine. In this study, the extract induced cytotoxic effect in a dose dependent manner and almost half of the cells were dead in 24 h at doses of 150 and 200 µg/mL.

It has been demonstrated that inflammation plays an important role in tumorgenesis and clonal evolution of cancer through stimulation of mutation and also it has effects on resistant of cancer cells after chemotherapy due to clonal evolution caused by mutation (1, 3, 5 and 15). The use of compounds with anti-inflammatory and anti-oxidant properties is an important strategy for cancer treatment because they can prevent more mutations caused by inflammation and lead to alleviate genomic instability and clonal evolution (1, 21). In addition, it has been suggested that medicinal plants may have chemopreventive activities (22). Previous reports have demonstrated that *satureja khuzestanica *has potent anti-inflammatory and antioxidant properties (11-13).

The cancer cells usually prohibit the apoptotic program through increased expression of anti-apoptotic proteins such as Bcl-2 or BclXL or by decrease in expression of pro-apoptotic proteins such as Bax and Bak (3). Our study showed that SKE at doses of 150 and 200 µg/mLcould increase the ratio of Bax/Bcl-2 proteins expression. The accumulation of pro-apoptotic proteins such as Bax and Bak in the mitochondrial membrane lead to release proteins such as cytochrome c and apaf-1 into cytoplasm. After that, apoptosome complex can be formed and eventually leads to produce activated form of caspase 9. This caspase activates executive procaspases such as 6, 7, and 3 which promote the programmed cell death. Caspase 3 is the most important executive caspase and its activation confirms that apoptosis has been certainly done (23). In this study, it was shown that SKE properly activates caspase 3 after 24 h incubation in MCF-7 cancer cells. Due to the severity of the caspase 3 cleavage and the Bax/Bcl-2 ratio , it can be concluded that possibly other mitochondrial pro-apoptotic and antiapoptotic proteins such as bclxl and Bak are involved in inducing apoptosis program by SKE. For the first time, it has been shown that SKE induces apoptosis and it has anticancer properties. The results largely match with the results of previous research on carvacrol (16, 17) and also, the presence of carvacrol in satureja extract probably plays a main role in the induction of apoptosis. However, we have previously reported that *satureja khuzestanica* extract can prevent apoptosis in high glucose-treated PC12 cells (11). It seems that the extract specifically kills cancer cells.

Cell cycle control is deregulated in cancerous situation and cancer cells in spite of defects in their genome can easily pass cell check points thus continuing to grow and proliferate. Cancer cells often over-express cell cycle proteins such as cyclin D1 and drive the cycle to proliferation without any interruption. Cyclin D1 plays an important role in passing of cell cycle through the S phase to G phase and also, reduced expression of cyclin D1 causes cell cycle arrest at the G 1 phase (3, 24). SKE at doses of 150 and 200 could reduce the protein expression of cyclin D1 in MCF-7 cancer cell thus causing reduction in the growth and proliferation of these cells. It has been demonstrated that the expression of cyclin D1 is associated with activities of more than 100 such proteins in DNA repair, transcription regulation, cell structure, proliferation, and other proteins (24). Therefore, the drugs helping to reduce the expression of cyclin D1 in cancer cells will have a significant impact on the intracellular processes of cancer and can be useful in treatment and prevention of cancer. However, immunoblotting detection of cyclin D1 concurrent with the evaluation of cell cycle arrest needed to approve the effect of SKE on cancer treatment.

Vincristine is now widely used in chemotherapy and prevents the formation of microtubules thereby preventing mitotic spindle formation and cell division progression. However, this medicine has side effects such as nausea and vomiting, stomach pain and cramps, constipation, diarrhea, jaw pain, headache, and other aches. In this study, it was shown that combination of low doses of vincristine and SKE significantly increases cytotoxic rate and has a synergic effect on breast cancer cell line MCF-7. Increasing the rates of cleavage caspase 3 and expression of cyclin D1 were also observed in combination therapy. 

The data showed that SKE and vincristine has synergic effect on apoptosis and cell cycle markers which is important for cancer fighting. Besides dosage reduction of chemotherapy medicines along with promoting their action by safe and without side effects compounds cause increased patient satisfaction and more effective treatment.

*Satureja khuzestanica* as vegetable is used as flavoring and its analgesic effect in southern of Iran. Due to the antioxidant and anti-inflammatory effect of *satureja khuzestanica* and also new results of our present study this plant has a good potential for further and more complete investigation on the mechanisms related to cancer treatment and prevention.
